# The Class III Peroxidase Encoding Gene *AtPrx62* Positively and Spatiotemporally Regulates the Low pH-Induced Cell Death in *Arabidopsis* *thaliana* Roots

**DOI:** 10.3390/ijms21197191

**Published:** 2020-09-29

**Authors:** Jonathas Pereira Graças, Philippe Ranocha, Victor Alexandre Vitorello, Bruno Savelli, Elisabeth Jamet, Christophe Dunand, Vincent Burlat

**Affiliations:** 1 Escola Superior de Agricultura “Luiz de Queiroz”, University of São Paulo, São Paulo 13418-900, Brazil; 2 Laboratoire de Recherche en Sciences Végétales, Université de Toulouse, CNRS, UPS, 24 chemin de Borde Rouge, 31320 Auzeville-Tolosane, France; ranocha@lrsv.ups-tlse.fr (P.R.); savelli@lrsv.ups-tlse.fr (B.S.); jamet@lrsv.ups-tlse.fr (E.J.); dunand@lrsv.ups-tlse.fr (C.D.); 3 Centro de Energia Nuclear na Agricultura, University of São Paulo, São Paulo 13400-970, Brazil; victor@cena.usp.br

**Keywords:** acidic stress, *Arabidopsis thaliana*, cell mortality, cell wall, cell wall remodeling, root tip, root zone, ROS homeostasis, superoxide depletion

## Abstract

Exogenous low pH stress causes cell death in root cells, limiting root development, and agricultural production. Different lines of evidence suggested a relationship with cell wall (CW) remodeling players. We investigated whether class III peroxidase (CIII Prx) total activity, CIII Prx candidate gene expression, and reactive oxygen species (ROS) could modify CW structure during low pH-induced cell death in *Arabidopsis thaliana* roots. Wild-type roots displayed a good spatio-temporal correlation between the low pH-induced cell death and total CIII Prx activity in the early elongation (EZs), transition (TZs), and meristematic (MZs) zones. In situ mRNA hybridization showed that *AtPrx62* transcripts accumulated only in roots treated at pH 4.6 in the same zones where cell death was induced. Furthermore, roots of the *atprx62-1* knockout mutant showed decreased cell mortality under low pH compared to wild-type roots. Among the ROS, there was a drastic decrease in O_2_^●−^ levels in the MZs of wild-type and *atprx62-1* roots upon low pH stress. Together, our data demonstrate that *AtPrx62* expression is induced by low pH and that the produced protein could positively regulate cell death. Whether the decrease in O_2_^●−^ level is related to cell death induced upon low pH treatment remains to be elucidated.

## 1. Introduction

Around 70% of arable soils are acidic (pH < 5.5) [1]. In these soils, a combination of unfavorable factors occurs for plant development, such as mineral toxicity and low nutrient level, especially for cations such as calcium [2]. These factors contribute to decreased root growth with a worldwide negative impact on crop productivity [3]. A major part of acidic soils is encountered in developing countries that are distributed in tropical and subtropical zones in which the economy is highly dependent on agricultural production [4]. Thus, besides the scientific issue, understanding how acidity affects plant growth is highly relevant for agriculture and food production safety.

In addition to the abovementioned factors present in acidic soils, exogenously applied low pH conditions, such as those achieved by the addition of HCl in plant growth solutions, are used in laboratory assays. It simulates soil stressful conditions, causing root growth inhibition in relevant dicot crops, such as *Solanum lycopersicum* [5], monocots, such as *Triticum aestivum* [6] or model plants such as *Arabidopsis thaliana* [2]. The root growth arrest upon low pH is suggested to be an active plant response to lessen cell death [5]. Cell death was reported within minutes of low pH treatment [2] and was consistently observed in growing root hair cells [7], lateral root tips [2], primary root tip cells in *A. thaliana* [8,9] or secondary root tips such as in *S. lycopersicum* [5]. Root cells are originating from cell division in the meristematic zone (MZ) [10]. The cell wall (CW) structure and composition are modified in the transition zone (TZ) where cells grow isodiametrically, prior to undergo a massive polarized increase in size in the elongation zone (EZ) [10,11]. The level of expression of genes encoding several CW-related enzymes, including class III peroxidases (CIII Prxs) is increased in EZ [12]. Finally, after reaching their full size, the epidermis trichoblast cells start to differentiate and form root hairs which elongate through tip growth [10].

While exposed to low pH, young developing root hairs either burst or stop their tip growth [7,13]. One hypothesis is that low pH may cause cell wall disturbances (CWDs), such as an excessive loosening, leading to root hair bursting. Conversely, the arrest in root hair elongation upon low pH might indicate the capacity of the CW to stiffen, thus protecting the structure from proton action and, therefore, preventing bursting. The cell wall of excised lateral roots of *Pisum sativum* apparently loosened upon low pH treatment [14]. In root tip tissues, the cell mortality due to the low pH is alleviated with an increase in calcium concentration within culture medium [2,8]. Calcium plays crucial roles in stabilizing an important class of CW polysaccharides, the homogalacturonans [15], as well as for the maintenance of CW integrity (CWI) [16]. One interpretation is that increased calcium decreases low pH-dependent CWDs, in turn suppressing the low pH-induced cell death. Transcription factors of the SENSITIVE TO PROTON RHIZOTOXICITY (STOP) family control the expression of genes related to CW integrity and are crucial for low pH tolerance in roots [17]. The cell wall-related genes were among those which had expression levels that were the most affected by low pH stress in *A. thaliana* [18]. Recently, it was reported that increase in CWDs, such as loosening, precedes cell death due to the low pH in TZ [19]. From the above cited reports, it seems that low pH toxicity is related to CW. Thus, CW-related players are likely to be involved in cellular responses of roots cells to low pH stress.

Among the CW-related players, CIII Prxs (E.C.1.11.1.7) play crucial roles during root CW remodeling [12,20]. These modifications are required for cell elongation and consequent root growth [10] and also occur as a response to constraints [20]. Most of the 73 *A. thaliana* CIII Prxs are expressed in roots and secreted to the apoplast [21]. They play biological roles in production and metabolism of reactive oxygen species (ROS) in the apoplast [20,22]. Upon exogenous low pH treatment, the expression of 13 *CIII Prx* genes was shown to be differentially regulated in *A. thaliana* roots [18]. In addtion, mild or strong low pH stress increased CIII Prx activity in *S. lycopersicum* roots [5]. These reports suggest that *CIII Prx* genes are regulated either transcriptionally or post-transcriptionally in roots exposed to low pH stress. Thus, our hypothesis is that individual CIII Prx isoforms might play a direct role in cell mortality in roots upon exogenous low pH stress.

The ROS produced by nicotinamide adenine dinucleotide phosphate (NADPH) oxidases are crucial for the formation and maintenance of CWI [23,24]. The oxido-reductase activity of CIII Prxs can either promote loosening or stiffening of the CW, and this activity consumes hydrogen peroxide (H_2_O_2_) as an electron donor [25]. Superoxide ions (O_2_^●−^) can be produced by NADP (H) oxidation or CIII Prx activity and O_2_^●−^ can also be converted spontaneously or by superoxide dismutase to H_2_O_2_ [20,25]. During the peroxidative cycle, CIII Prxs consume H_2_O_2_ and superoxide ions (O_2_^●−^) to generate hydroxyl radicals (^●^OH), which by themselves can promote CW loosening [26]. Thus, in concert with NADPH oxidases, CIII Prxs are key players which control ROS levels in the apoplast. Prominently, the ratio between H_2_O_2_ and O_2_^●−^ is finely adjusted spatially in the different root zones by CIII Prx activity for maintenance or arrest of growth [27,28].

Reactive oxygen species homeostasis is tightly regulated by several intracellular [29] or apoplast actors [22] to control their potential cellular toxicity. Peanut roots treated with low pH and Al³^+^ displayed increased ROS production causing cell death that was alleviated by addition of ROS scavengers [30,31]. Inhibition of total CIII Prx activity decreased ROS levels and sensitivity of barley roots to low pH and aluminum, the latter being toxic only in acidic conditions [32]. On the other hand, inhibition of NADPH oxidase and CIII Prx activity in *S. lycopersicum* greatly increased the sensitivity of root cells solely to low pH [5]. Taking these cited reports into account, it seems that depending on the type and magnitude of the acidic stress, ROS and CIII Prx activity can modulate cellular sensitivity to low pH.

Out of the 73 CIII Prxs predicted to be encoded in the genome of *A. thaliana*, 38 isoforms were detected in roots by proteomic analysis [33]. Hence, to identify which isoform(s) could be responsive to exogenous low pH stress and/or how they could regulate ROS homeostasis during this stressful seeming challenging. This issue has been tackled upon by acidic stress alone [5] or in combination with aluminum stress [32]. However, these studies were usually performed through pharmacological approaches, applying inhibitors of CIII Prxs or NAPDH oxidases [5,32]. These pharmacological studies are useful to quickly block enzymatic activities or ROS production. However, pharmacological inhibitors show two well-known disadvantages: cell toxicity or undesirable side-effects upon other metabolisms. So far, it is not clear in which root zones CIII Prx activity is induced by low pH or whether it has spatial coincidence with the low pH-induced cell death. Importantly, studies investigating the role of specific CIII Prx isoforms are lacking so far.

In this article, we show that CIII Prx activity and disruption in ROS balance are spatiotemporally correlated with low pH-induced cell death in roots of *A. thaliana*. Furthermore, by combining data mining of previously published transcriptomics data and reverse genetics tools, we were able to show that among the 73 CIII Prx encoding genes, *AtPrx62* expression is induced in low pH-induced cell death zones and could be necessary for this low pH sensitive response.

## 2. Results

### 2.1. Cell Death, CIII Prx Activity and ROS Distribution Colocalized in Wild-Type Roots Exposed to Low pH

First, we examined in wild-type roots (Col-0) exposed to low pH whether a spatial correlation occurred between (i) cell death (monitored through Evans blue staining), (ii) CIII Prx activity (visualized by guaiacol/H_2_O_2_), and (iii) ROS (O_2_^●−^ and H_2_O_2_) distribution (stained with nitro blue tetrazolium chloride (NBT) and hydroxyphenyl fluorescein (HPF), respectively). The guaiacol/H_2_O_2_ assay, used to visualize the CIII Prx activity, allows the detection of the total endogenous activity without distinction among the isoforms.

**Figure 1:**
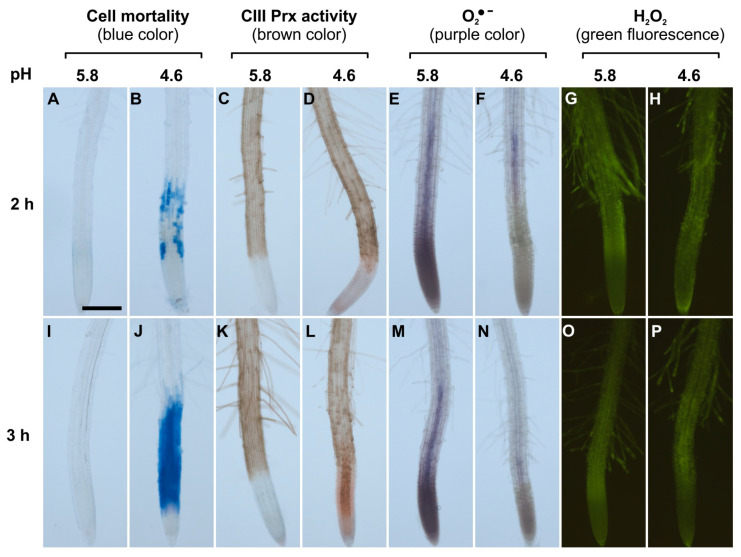
Spatio-temporal correlation between cell mortality, class III peroxidase (CIII Prx) activity, and ROS distribution in roots of *Arabidopsis thaliana* Col-0 upon low pH stress. Col-0 seedlings were treated at pH 5.8 (**A**,**C**,**E**,**G**,**I**,**K**,**M**,**O**) or 4.6 (**B**,**D**,**F**,**H**,**J**,**L**,**N**,**P**) for 2 h (upper panels) or 3 h (lower panels). Cell mortality was examined staining with Evans blue. Endogenous CIII Prx activity (total activity without distinction among the isoforms) was detected with a guaiacol/H_2_O_2_ assay. The detection of O_2_^●−^ was performed using nitro blue tetrazolium chloride (NBT). The detection of H_2_O_2_ was realized using hydroxyphenyl fluorescein (HPF). Scale bar: 200 μm. Three independent experiments (*n* = 10) were performed with similar results and representative images are shown. Details of root zones are presented in Supplementary Materials Figure S1.

In control roots treated at pH 5.8 for 2 or 3 h, cell death did not occur (Figure 1A,I) and CIII Prx activity was detected along the entire root except in MZs (Figure 1C,K; Supplementary Materials Figure S1B). Roots treated at pH 4.6 for 2 h showed cell death in the TZ and early EZ as indicated by the blue color of Evans blue uptake, and this cell death progressed toward the MZ after 3 h of low pH treatment (Figure 1B,J; Supplementary Materials Figure S1D). The treatment at pH 4.6 sequentially increased CIII Prx activity in the TZ and then in the MZ, with a stronger signal observed in the stele (Figure 1D,L; Supplementary Materials Figure S1C). Prominently, the CIII Prx activity increase in the TZ and MZ occurred before the death of cells in these zones (Figure 1D; Supplementary Materials Figure S1).

The accumulation of superoxide ion (O_2_^•−^) was visualized with NBT which forms a purple formazan precipitate after oxidation by O_2_^•−^ [27]. Control roots treated at pH 5.8 for 2 or 3 h accumulated O_2_^•−^ mostly in MZ, as compared to TZ and EZ (Figure 1E,M; Supplementary Materials Figure S1A). This pattern was similar to the previously observed O_2_^•−^ distribution in *A. thaliana* root MZ [27]. In sharp contrast, roots treated at pH 4.6 for 2 or 3 h showed no signal for O_2_^•−^ accumulation in MZ (Figure 1F,N). Thus, upon low pH, the O_2_^•−^ distribution in the root tip was disrupted. This result could not be interpreted as a direct effect of low pH on NBT staining, since the reaction was performed after low pH treatment in phosphate buffer at pH 6.1 [27].

The H_2_O_2_ distribution in roots was examined with hydroxyphenyl fluorescein (HPF) which becomes fluorescent after oxidation by H_2_O_2_ and peroxidases [27]. Control roots treated at pH 5.8 or 4.6 for 2 or 3 h showed no detectable difference in H_2_O_2_ distribution. The fluorescence was similar and pronounced in TZ, EZ, and root hairs (Figure 1G,H,O,P), similarly to previous report [27]. The hydrogen peroxide level was also quantified by a highly sensitive fluorometric assay employing Amplex^®^ Red. Only a slight decrease in H_2_O_2_ levels could be observed after a 4 h treatment in roots at pH 4.6 as compared to pH 5.8 (Supplementary Materials Figure S2).

### 2.2. Data Mining of Published Transcriptomics Data Searching for CIII Prx Genes Expression upon Low pH Treatment: Identification of Candidate Genes of Interest

**Figure 2:**
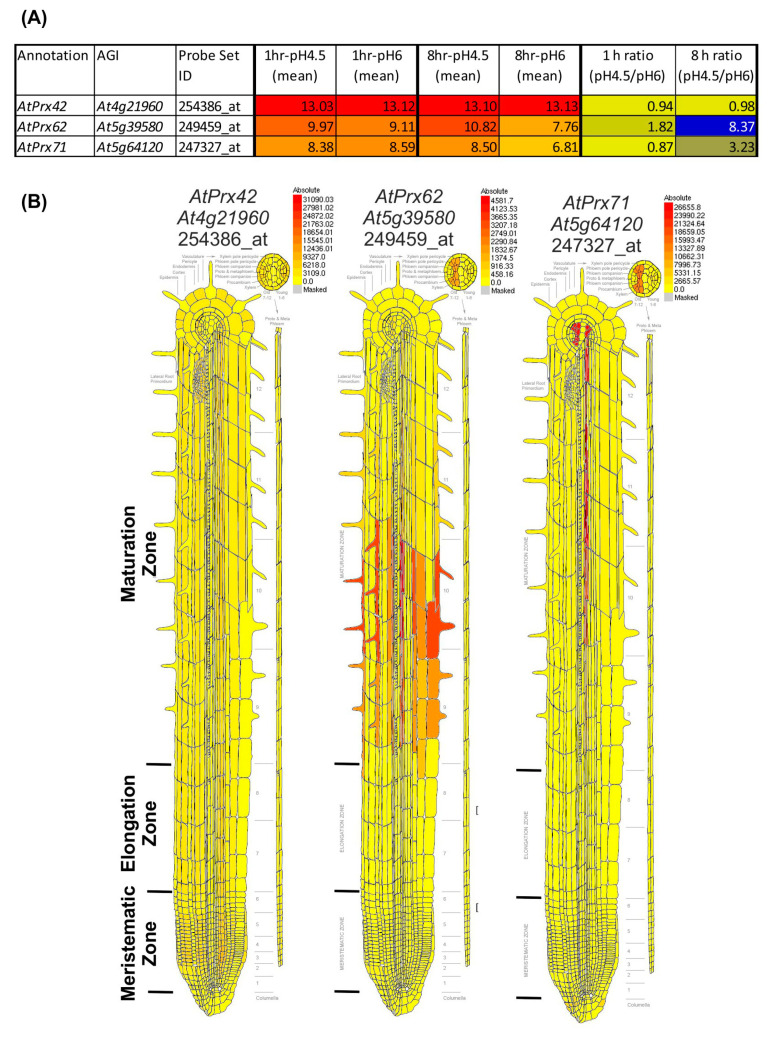
Transcriptomic data mining enables selecting *AtPrx62* and *AtPrx71* CIII Prx candidate genes for involvement in low pH response, and *AtPrx42* as a control gene. (**A**) NASC 470 low pH transcriptomic data [18] was downloaded and edited. The mean of the log2(expression values) for each condition (*n* = 3) was calculated as well as the ratio of absolute expression for low pH versus control pH. The 73 *CIII Prxs* [37] were searched within the transcriptomic data. An absolute heat map was drawn for the expression values (red to yellow to grey) with an arbitrary threshold value set as 5. An absolute heat map was also drawn for log2 (ratio) (blue to yellow). Note that *AtPrx62* displayed the highest ratio among the 73 *CIII Prxs*. *AtPrx71* was selected as a second candidate for its intermediate ratio despite its lower absolute expression values whereas *AtPrx42* was chosen as a control considering its strong constitutive expression (see Supplementary Materials Table S1 for full data and phylogenetic grouping). (**B**) Tissue-specific expression map of the three selected genes from the electronic fluorescent pictographic (eFP) browser [38]. Note that the absolute maximum expression values are different for each gene.

Our results showed a spatio-temporal correlation between the occurrence of cell death, the increase in CIII Prx activity, and the decrease of O_2_^●−^ upon low pH stress. Thus, we took advantage of a publicly available low pH transcriptomic dataset from *A. thaliana* roots [18] to search among the 73 CIII Prx-encoding genes—those that were the most induced after 1 or 8 h of low pH treatment. Among them, *AtPrx62* (*At5g39580*), encoding an *A. thaliana* CIII Prx from the phylogenetic group 2 [34], was the best candidate since its expression was induced 8.37 fold after 8 h of low pH treatment (Figure 2A; Supplementary Materials Table S1 for full data and phylogenetic grouping). According to published tissue-specific transcriptomics [35], *AtPrx62* is expressed at high levels in epidermal and stele cells at the beginning of the maturation zone in which root hairs start tip-growth (Figure 2B) close to the TZ in which low pH-induced cell death occurred (Figure 1I,J). A second-level candidate was *AtPrx71 (At5g64120)* encoding another group 2 CIII Prx closely phylogenetically related to *AtPrx62* (Supplementary Materials Table S1) showing a 3.23 fold induction of expression, but with a lower expression level than *AtPrx62* (Figure 2A) and a more distal expression pattern (Figure 2B). Finally, *AtPrx42 (At4g21960)* (phylogenetic group 1) was selected as a control considering its strong constitutive expression pattern (Figure 2; Supplementary Materials Table S1). Thus, we examined the sensitivity to low pH of mutants impaired in *AtPrx62* or *AtPrx71* [36].

### 2.3. Cell Viability and Total CIII Prx Activity in *Atprx62* and *Atprx71* Mutants Exposed to Low pH

Although cell viability was not examined in Lager et al.’s [18] work, the low pH stress seemed to be less severe than in our conditions. Our treatment solution was based on a low ionic strength buffer and low calcium supply, important for a rapid imposition of low pH stress [2,5,9]. The expression of *AtPrx62* was markedly induced only after 8 h, rather than 1 h of stress treatment in Lager’s work (Figure 2A), when the stress exposure seemed to become critical. Thus, we extrapolated that our 2 to 3 h stress conditions (Figure 1) that roughly corresponded to the transcriptomics data obtained after 8 h rather than 1 h of low pH treatment in the Lager et al. work [18].

**Figure 3:**
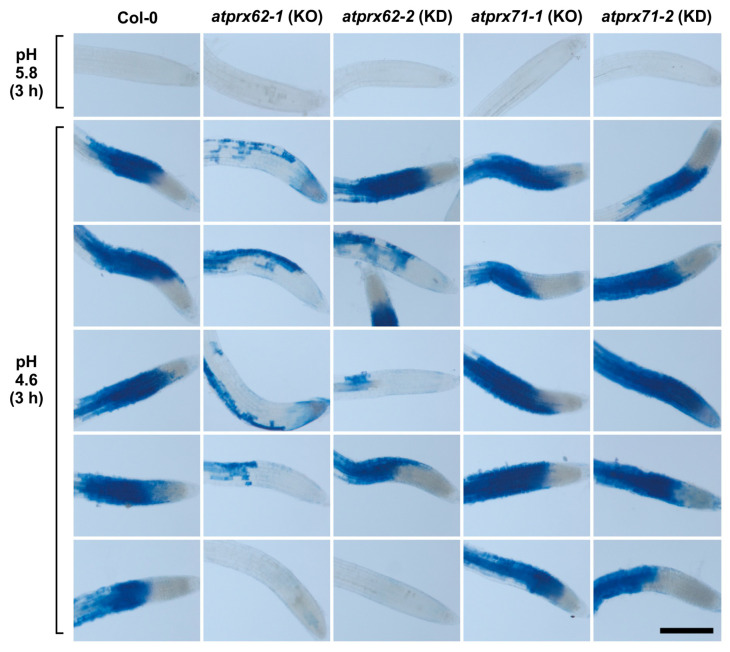
Analysis of low pH-induced cell death phenotypes in *atprx62* and *atprx71* mutants treated with strong low pH stress for 3 h. Roots treated at pH 5.8 or 4.6 for 3 h were stained with Evans blue. The blue color indicates cell mortality. From left to right: Col-0, *atprx62-1* KO mutant, *atprx62-2* KD mutant, *atprx71-1* KO mutant, *atprx71-2* KD mutant. KO: knockout; KD: knockdown; Scale bar: 200 µm. This initial screening was performed with 10 biological replicates (roots from individual seedlings). From at least 13 roots, five representative images are shown for each genotype upon pH 4.6. Note that only *atprx62-1* displayed a clear reduced cell mortality phenotype. For simplification, one image is shown for each genotype upon pH 5.8 (control), but in none of the genotypes was there cell death in these control root repeats.

As a first screening step, we applied our low pH stress conditions (3 h at pH 4.6) to seedlings of the *atprx62* and *atprx71* mutants. Then, Evans blue staining was performed (Figure 3). Interestingly, only the *atprx62-1* knockout (KO) mutant [36] showed a clear reduced cell mortality phenotype as compared to Col-0 (Figure 3). Indeed, the *atprx62-1* knockdown (KD) mutant, that displayed residual *AtPrx62* expression (40–50%) [36], only displayed a tendency of reduction of cell mortality whereas both *atprx71-1* KO and *atprx71-2* KD mutants [36] displayed a pattern similar to that of Col-0 (Figure 3).

**Figure 4:**
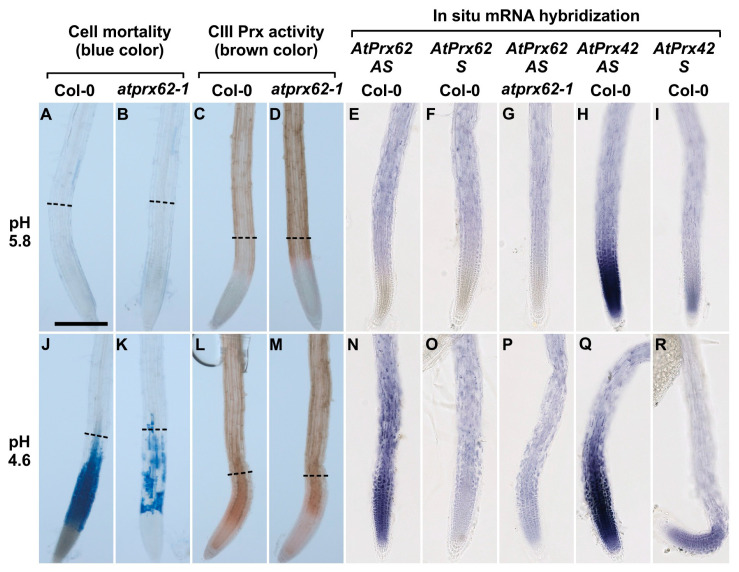
Low pH dependent spatio-temporal correlation between cell mortality, CIII Prx activity, and *AtPrx62* expression in *Arabidopsis thaliana* roots. Col-0 (**A**,**C**,**E**,**F**,**H**,**I**,**J**,**L**,**N**,**O**,**Q**,**R**) and *atprx62-1*(KO mutant) (**B**,**D**,**G**,**K**,**M**,**P**) roots treated at pH 5.8 (**A**–**I**) or pH 4.6 (**J**–**R**) for 2 h were stained with Evans blue for cell mortality (A,B,J,K), stained with guaiacol/H_2_O_2_ for CIII Prx activity (**C**,**D**,**L**,**M**) or hybridized with *AtPrx62* digoxygenin-labeled antisense (AS) or sense (S) probes used as negative controls (**E–G, N–P**). Positive control with the *AtPrx42* probes was performed as labeled (**H**,**I**,**Q**,**R**). The dashed lines (**A**–**D**; **J**–**M**) show the upper limit of the regions of interest (ROIs) used for quantification on biological repeats (see Figure 5). Scale bar: 200 µm. For cell mortality and CIII Prx activity, three independent experiments were performed with similar results and representative images are shown (**A**–**D**; **J**–**M**). For in situ hybridization, two independent experiments with 25 biological replicates (roots) were performed with similar results and representative images are shown (**E**–**I**; **N**–**R**). Note that the spatiotemporal correlation between the low pH-induced cell mortality (**J**), CIII-Prx activity (**L**), and *AtPrx62* expression (**N**) occurring around the transition zone in Col-0 was strongly reduced or lost in *atprx62-1* (**K**,**M**,**P**). Note also that the specificity of the *AtPrx62* expression pattern (**N**) was attested by comparison with the similar background signals observed with the *AtPrx62* sense negative control probe in Col-0 (**O**) and *AtPrx62* antisense probe in *atprx62-1* (**P**). Finally, note that the specificity of the induction of *AtPrx62* expression upon low pH treatment (**E**,**N**) is reinforced by the constitutive expression of *AtPrx42* observed with the *AtPrx42* antisense probe (**H**,**Q**).

**Figure 5:**
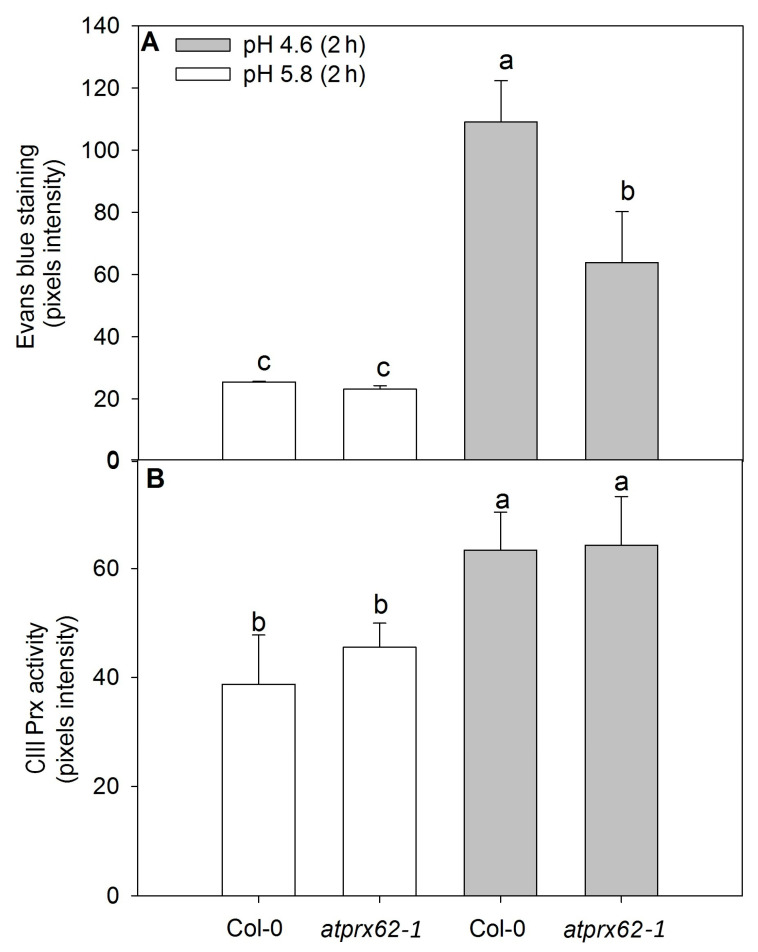
Comparison of cell mortality and CIII Prx activity in roots of *Arabidopsis thaliana* Col-0 and the *atprx62-1* (KO mutant) treated at pH 5.8 or 4.6 for 2 h. The pixels from the images were counted in regions of interest extending up to 500 µm from root tips stained with Evans blue (cell death) or up to 350 µm from root tips stained with a guaiacol/H_2_O_2_ assay (CIII Prx activity) (see dashed lines in Figure 4A–D, J–M for examples). (**A**) The increase in pixels’ intensity indicates an increase in cell death. (**B**) The increase in pixels intensity indicates an increase in CIII Prx activity. The bars are the standard error of three independent experiments. Different letters (a, b and c) indicate significant differences Statistical analysis was performed by Duncan’s test. Note the good correlation between low pH-induced cell death and CIII Prx activity. Note that the decreased cell death observed in *atprx62-1* as compared to WT was not followed by a reduction in CIII Prx activity.

Therefore, we proceeded to further analyze the *atprx62-1* KO mutant. The roots of the genotypes Col-0 and *atprx62-1* treated at pH 5.8 repeatedly showed viable cells with negligible Evans blue staining (Figure 4A,B and Figure 5A). As expected, Col-0 roots treated at pH 4.6 showed increased cell mortality in TZs and MZs (Figure 4J and Figure 5A). However, *atprx62-1* roots were significantly less sensitive to pH 4.6 than Col-0 roots as indicated by decreased Evans blue uptake (Figure 4K and Figure 5A).

The CIII Prx activity showed similar patterns in the roots of Col-0 and *atprx62-1* treated at pH 5.8 (Figure 4C,D and Figure 5B). However, Col-0 roots showed an increase in CIII Prx activity when treated at pH 4.6 for 2 h (Figure 5B), with higher staining in the region that seemed to be stele cells (Figure 4L, Supplementary Materials Figure S3). In the *atprx62-1* KO mutant, there was also a slight increase in CIII Prx activity in MZ and TZ compared to roots treated at pH 5.8 (Figure 4M, Figure 5B, Supplementary Materials Figure S3). 

No difference in O_2_^●−^ distribution was observed in *atprx62-1* roots as compared to Col-0 roots. Both genotypes displayed similar patterns of reduced O_2_^●−^ labeling in MZs with NBT following low pH treatment (Supplementary Materials Figure S4).

### 2.4. Tissue-Specific Expression Patterns of *AtPrx62* and *AtPrx42* in Roots upon Low pH Treatment 

We next localized *AtPrx62* expression through whole mount in situ hybridization. No significant specific signal was detected in Col-0 roots treated at pH 5.8 and hybridized with the *AtPrx62* antisense probe (Figure 4E; Supplementary Materials Figure S5) when compared with hybridization with the *AtPrx62* sense probe used as a negative control (Figure 4F; Supplementary Materials Figure S5). The lack of specific signal was also observed in *atprx62-1* treated at pH 5.8 and hybridized with the *AtPrx62* antisense probe (Figure 4G; Supplementary Materials Figure S5).

Interestingly, Col-0 roots treated at pH 4.6 and hybridized with *AtPrx62* antisense probe showed a significant signal in TZ, MZ, and early EZ (Figure 4N; Supplementary Materials Figure S5) when compared with the sense probe hybridization (Figure 4O; Supplementary Materials Figure S5). This signal was coincident with the zone of cell death (Figure 4J; Supplementary Materials Figure S5). The lack of signal was also confirmed in roots of *atprx62-1* treated at pH 4.6 and hybridized with the antisense probe for *AtPrx62* (Figure 4P; Supplementary Materials Figure S5). The specificity of the *AtPrx62* expression pattern at pH 4.6 was strengthened by the observation of a constitutive expression pattern for *AtPrx42* (Figure 4H,I,Q,R; Supplementary Materials Figure S5). These results corroborated, from a spatiotemporal point of view, the transcriptomics values and ratios (Figure 2A). Importantly, the in situ hybridization has allowed demonstrating the spatiotemporal correlation between the low pH-induced *AtPrx62* expression and cell death zone (MZ, TZ, and early EZ) upon low pH treatment, thus suggesting that *AtPrx62* was involved in low pH-induced cell death.

## 3. Discussion

The cell wallremodeling players are involved in low pH-induced sensitivity responses such as arrest in root growth or cell mortality in roots [2,5,9,17,18]. Class III peroxidases (CIII Prxs) and ROS are remarkable CW remodeling players [20,25]. However, information about their involvement with low pH-induced cell death is missing. Altogether, our results show a spatiotemporal correlation in *A. thaliana* roots, between low pH-induced cell death, CIII Prx activity, *AtPrx62* expression, and ROS distribution.

### 3.1. AtPrx62 Expression Is Spatiotemporally Correlated to Low pH-Induced Cell Death in Roots

We mined previously published transcriptomic data [18] to find *CIII Prx* gene candidates induced during low pH stress that might be possibly involved in low pH-induced cell death. Among the 73 *CIII Prx* genes predicted in the *A. thaliana* genome [21], the involvement of *AtPrx62* in low pH-induced cell death was examined, because it is the CIII Prx encoding gene with the highest induction of its expression (8.37 fold) upon low pH treatment (Figure 2A, Supplemental Materials Table S1).

**Figure 6:**
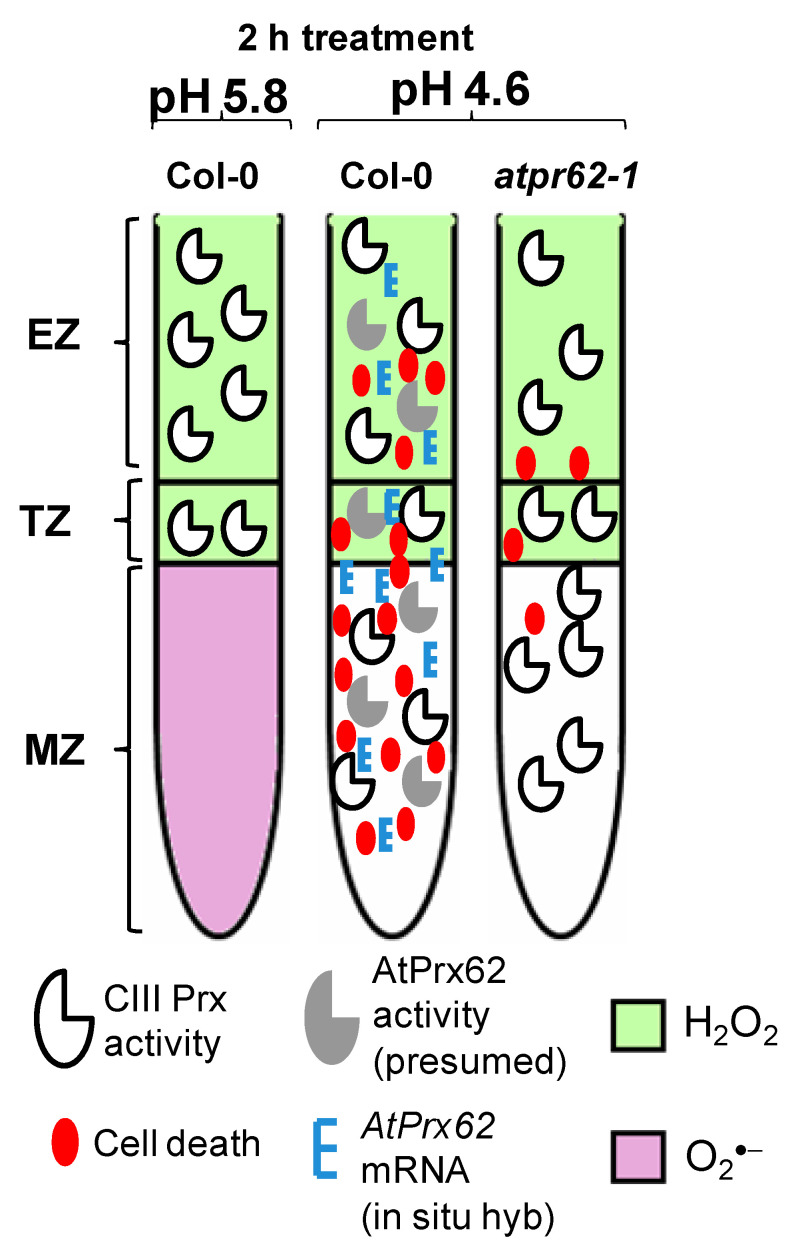
Spatio-temporal model proposed for the action of AtPrx62 in roots of *A. thaliana* upon low pH and the progression of cell death. In Columbia-0 (Col-0) roots, a reliable spatio-temporal correlation was observed in EZs, TZs, and MZs between low pH-induced CIII Prx activity (total activity: without distinction among the isoforms), *AtPrx62* mRNA distribution, and cell death. The low-pH-induced cell death and *AtPrx62* mRNA accumulation were highly decreased in roots of *atprx62-1* KO mutant indicating that *AtPrx62* was positively involved in the progression of low pH-induced cell death. However, no decrease could be measured for the CIII activity in *atprx62-1*. The prominent disruption in H_2_O_2_/O_2_^●−^ balance in root tips upon low pH stress was not dependent on *AtPrx62* gene products. Whether the observed decrease in O_2_^●−^ is linked to cell death upon low pH or is caused by arrest of its production or by exacerbated scavenging, remains to be elucidated.

Our results indicate that *AtPrx62* spatiotemporal expression is positively correlated to the low pH-induced cell death in MZ, TZ, and early EZ as described in our model (Figure 6). The most compelling indications for this are as follows: (i) the expression of *AtPrx62* in low pH-treated Col-0 roots increased in TZ, MZ, and early EZ, and this was correlated with the observed pattern of cell death upon low pH stress; (ii) cell death was greatly suppressed in MZ, TZ, and early EZ of the *atprx62-1* KO mutant treated at pH 4.6 compared to Col-0, despite the increase in total CIII Prx activity at pH 4.6 observed in these zones for both Col-0 and *atprx62-1*.

Class III peroxidases belong to a large family dedicated to CW remodeling with 38 isoforms identified in the *A. thaliana* root CW proteome [33]. These proteins could play either specific and complementary roles (loosening or stiffening) with possible functional redundancy [20]. It is thus challenging to investigate the biological function of specific isoforms. Thus, it was rather remarkable to find that a KO mutant in a single CIII Prx isoform (AtPrx62) caused an effect on cell mortality due to the fact of low pH. A KO mutation in *AtPrx71*, the gene with the third most induced expression (3.23 fold) upon 8 h of low pH stress (Figure 2A; Supplementary Materials Table S1) did not result in any significant difference with respect to cell death upon low pH compared to Col-0. Hence, our study illustrates the importance of reverse genetic studies to uncover the functions of CIII Prxs [20]. In situ hybridization has allowed refining the tissue-specific expression pattern of this gene. Indeed, while tissue-specific transcriptomics argued for *AtPrx62* expression in early MZ (Figure 1B), our results clearly showed that the low pH-induced *AtPrx62* expression occurred below this zone in MZ, TZ, and early EZ, i.e., in the zones where low pH-induced cell death occurred (Figure 4 and Figure 6), thus suggesting that *AtPrx62* was involved in low pH-induced cell death.

Intriguingly, *AtPrx62* does not seem to be regulated by SENSITIVE TO PROTON RHIZOTOXICITY 1 (STOP1) [39], a transcription factor involved in low pH and Al^3+^ tolerance [8,39,40]. In the same way, *AtPrx62* is not a direct target of UPBEAT1, a transcription factor that regulates the expression of other *CIII Prx* genes necessary to control the balance between H_2_O_2_ and O_2_^●−^ in root tips controlling root growth [28]. However, *AtPrx62* expression was upregulated in *A. thaliana* roots after 6 h of aluminum stress [41] which is appreciably toxic for roots at low pH. Unfortunately, cell death was not assessed in the quoted report, neither the gene expression level in a control at a pH higher than 5.0.

Although we have shown the involvement of *AtPrx62* in cell death, we do not know yet if upon low pH stress, the presumed AtPrx62 activity in the apoplast contributes to CWDs. The cell wall disturbances seem to be relevant for the sensitive responses due to the low pH in roots [7,17,19]. With excessive loosening, it is a CWD that causes CW yielding in root hairs upon low pH [7]. Hence, if the presumed AtPrx62 activity causes loosening of the root CWs upon low pH, it could accelerate CWDs upon the stress. Recently, it was shown that in seed endosperm, *AtPrx62* belongs together with *AtPrx69*, *AtPrx16,* and *AtPrx71* to a *CIII Prx* co-expression cluster that could contribute to the stiffening of endosperm CW domains to control seed envelop rupture during early germination steps [36]. However, among these four genes, only *AtPrx62*, and to a lesser extent *AtPrx71* were found to be induced by low pH stress [18].

The CIII Prxs can regulate ROS levels by oxidizing aromatic compounds from CW components leading to a stiffened CW structure [20]. Alternatively, they produce ROS which by themselves are able to break covalent bonds between CW polymers causing the loosening of the CW structure [20,42]. This later role is the most plausible explanation for the bursting of root hairs treated with low pH [7,13]. A decrease in CW stiffness was reported in epidermal TZ cells before the onset of cell death in this root zone [19]. Thus, if the enzymatic activity of AtPrx62 is related to CW loosening, it could exacerbate CWDs caused by low pH and also accelerate the progression of cell death (Figure 6). *AtPrx10* and *AtPrx71* were also up-regulated after 8 h of low-pH treatment with the ratio of induction in pH 4.5 compared to pH 6.0 of 4.36 and 3.23 fold, respectively, but with lower absolute expression values (Supplementary Table S1). AtPrx62 and AtPrx71 were found as being CW-targeted proteins in seed endosperm of *A. thaliana* in the region of the envelop rupture [36]. The expression of *AtPrx71* was induced after CWDs due to treatment with isoxaben [43]. However, we observed no low pH-induced cell death phenotype in the mutants. Thus, if the enzymatic activity of AtPrx62 exacerbated CWDs upon low pH, it was likely to have consequences on cell survival.

Beyond the above considerations about AtPrx62 and CWDs in low pH-treated roots, the progression of cell death in roots exposed to low pH was reported in a related work of our group as a result of a programmed cell death (PCD) mechanism [19]. Before being targeted to the apoplast, unfolded secreted proteins can accumulate in the endoplasmic reticulum causing stress that disturbs the most vital cellular functions and can activate PCD [44]. The class III peroxidase *AtPrx62* was shown to be upregulated upon endoplasmic reticulum stress [45]. The cell wall disturbances can produce fragments of pectin molecules called oligogalacturonides (OGs) [46] which can trigger plant immune response leading to cell death [47]. Root hairs are rich in pectin [48] and very sensitive to low pH [7]. Suspension-cultured cells of *A. thaliana* treated with OGs showed downregulation of *AtPrx62* [49]. This information seems relevant since exogenous low pH stress is assumed to modify pectin structure in roots [2]. Hypoxia stress, which is well known to induce PCD in roots, negatively regulates *AtPrx62* expression by the ethylene-responsive factor ERF73/HRE1 [50]. Hence, besides low pH stress, signaling pathways from stress situations that ultimately triggers PCD seem to regulate *AtPrx62* expression. Thus, alternatively, we cannot exclude that AtPrx62 might be part of an orchestrated network leading to cell death, rather indirectly acting as a player of ROS signaling pathway.

### 3.2. Low pH Disrupts the O_2_^●−^/H_2_O_2_ Homeostasis in Roots

The CIII Prx activity controls ROS homeostasis by reducing their impaired electrons while oxidizing CW components and, thus, changing, physically, CW properties [22,27,42]. Reactive oxygen species production is linked to several signaling processes such as stomata closure or developmental programs such as pollen tube formation, root hair tip-growth and CW architecture in plants [51]. Reactive oxygen species levels are tightly controlled in intracellular compartments [29] or in the apoplast [22]. High ROS concentration occurs due to exacerbated production or failure in scavenging and can cause oxidative stress damaging proteins, lipids and DNA [29,52]. Altogether, these damages targeted on key cellular macromolecules can trigger cell death [52].

In our study, we observed an interesting pattern of ROS distribution in low pH-treated roots. In Col-0, there was an increased CIII Prx activity in MZ and TZ, but decreased O_2_^●−^ levels in MZs. No change in H_2_O_2_ levels was found in TZs, EZs, and root hairs at pH 5.8 or 4.6, similarly to a previous report [27]. Cell death-induced by low pH coincidently occurred in MZs, TZs, and early EZs, before root hairs fully undergo the tip-growth. Hence, excessive ROS levels could not be associated with low pH-induced cell death in roots, as it could have been expected. The mitochondria-dependent release of ROS was interpreted as a trigger for PCD in response to aluminum stress and low pH treatment in peanut roots [31]. The inhibition of CIII Prx activity with SHAM decreased H_2_O_2_ production and cell death due to aluminum stress and low pH in barley roots [32]. Unfortunately, the responses to low pH alone could not be evaluated in the works cited above because of a lack of control at higher pH (>5.5), but perhaps aluminum induced distinct ROS activities in stressed roots compared to low pH alone, as reported here.

A balance between levels of O_2_^●−^ in MZ and H_2_O_2_ in TZ and EZ was shown to be accurately adjusted in the root tip through CIII Prx activity [27]. This balance coordinates the rate of cell division in MZ for normal root growth [28]. Low pH caused a striking decrease in O_2_^●−^ levels in MZ and early TZ within 2 h of treatment, and, this apparently preceded both the increase in CIII Prx activity and cell death, as reported here (Figure 6). It remains to be investigated whether a modification in the O_2_^●−^/H_2_O_2_ balance upon low pH in roots could trigger to a signaling process inducing cell death. Since the ratio between O_2_^●−^ and H_2_O_2_ is crucial for root development [27,28], this hypothesis seems plausible.

It was reported that the horseradish CIII Prx can consume H_2_O_2_ and O_2_^●−^ to produce ^●^OH in vitro [26]. We did not examine O_2_^●−^ distribution and CIII Prx activity in roots before the first 2 h of low pH stress, yet CIII Prx activity could explain the observed decrease in O_2_^●−^ in low pH-stressed roots. However, there is no experimental evidence to understand how CIII Prxs could perform this reaction in vivo. Furthermore, O_2_^●−^ distribution was not altered in the *atprx62-1* KO mutant roots treated at pH 4.6 compared to Col-0, indicating that AtPrx62 is likely not involved in the decrease of O_2_^●−^ levels upon low pH stress (Figure 6).

Altogether, our results suggest that AtPrx62 could be a positive and spatiotemporal regulator of cell death in root tip cells upon exogenous low pH stress. Our study further confirms that CW remodeling players such as CIII Prxs are crucial for the occurrence of cell death in response to low pH stress. The disruption of the H_2_O_2_/O_2_^●−^ homeostasis in roots upon exogenous low pH may be part of a complex cell death signaling network and must be further elucidated.

## 4. Materials and Methods

### 4.1. Plant Material and Growth Conditions

*Arabidopsis thaliana* (Col-0) and T-DNA insertion lines in Col-0 background (*atprx62-1* (GK_287E07, knockout (KO) line), *atprx62-2* (SALK_151762, knockdown (KD) line), *atprx71-1* (SALK_123643, KO line), and *atprx71-2* (SALK_121202, KD line)) were originally obtained from the European Arabidopsis Stock Center [53] and their KO and KD status was previously described [36].

Seeds of *A. thaliana* were sterilized with sodium hypochlorite solution (5%) for 10 min under stirring and then washed with distilled water four times. The seeds were then transferred to Petri dishes containing a modified Hoagland’s solution [9] with final pH adjusted to 5.8 and 0.8% agar. Macronutrients consisted of 6 mM KNO_3_, 1 mM MgSO_4_, 4 mM Ca(NO_3_)_2_, and 2 mM NH_4_H_2_PO_4_. Micronutrients were composed of 0.03 µM NiSO4, 14 µM ZnSO_4_, 20 µM H_3_BO_3_, 0.02 µM Na_2_MoO_4_, 0.02 µM CuSO_4_, 0.02 µM CoCl_2_, 30 µM FeSO_4_, and 20 µM MnSO_4_. 

For all low pH treatments, at least 10 five-day-old seedlings were incubated in 250 mL Erlenmeyer’s with 20 mL of treatment solution composed of 0.5 mM CaCl_2_ and 0.6 mM Homopipes buffer (homopiperazine-1,4-bis(2-ethanesulfonic acid)) upon gentle stirring. The constant growth temperature was 22 ° C and the light intensity was approximately 120 μE.m^−2^.s^−1^.

### 4.2. Evaluation of Total CIII Prx Activity and ROS Distribution in Roots Exposed to Low pH

The total activity of endogenous CIII Prxs in roots was probed using a guaiacol/H_2_O_2_ assay [54]. Before the experiments, 0.125 % *v*/*v* guaiacol (Fluka, Munich, Germany) diluted in 200 mM phosphate buffer (pH 6.1) and stored at 4 °C. For the reaction, fresh 30% H_2_O_2_ was added to the guaiacol solution to reach a final concentration of 1.65 mM and roots were immediately covered with this solution in glass Petri dishes kept in the dark. After 5 min of incubation, the roots were gently washed by adding abundant water to stop the reaction and were instantaneously imaged on bright-field in a Zeiss Axio Zoom.V 16 stereomicroscope (Göttingen, Germany). 

Superoxide (O_2_^●−^) was detected in roots using nitro blue tetrazolium chloride (NBT) [27]. A solution of NBT (2 mM) was prepared in 20 mM phosphate buffer (pH 6.1). The roots were covered with this solution in glass Petri dishes kept in the dark for 15 min and the reaction was stopped by adding water. Immediately, the roots were imaged as described above. 

H_2_O_2_ was detected in roots using hydroxyphenyl fluorescein (HPF) [27]. The final concentration was 5 μM HPF in 20 mM phosphate buffer pH 6.1. The staining of roots with this solution was done in the dark for 15 min. The reaction was stopped by washing the roots in 20 mM phosphate buffer (pH 6.1). Immediately, the roots were imaged using a Zeiss Axio Zoom.V 16 stereomicroscope coupled to a GFP long pass filter cube (excitation 485/12 nm and emission >515 nm). The fluorescence background in roots stained with phosphate buffer alone was negligible.

H_2_O_2_ was quantified using two methods. (i) measurement of freely diffusing H_2_O_2_: After treatments, 2 cm of the root tips were excised (3 independent experiments each using 10 seedlings) and incubated in plastic tubes containing 1 mL of solution composed of 50 µM Amplex^®^ Red (10-acetyl-3,7-dihydroxyphenoxazine, ampliflu™ red, Sigma, St. Louis, Missouri, USA) and 2 U/mL horseradish Prx for 10 min in the dark. Following, the reaction was immediately stopped by adding SHAM 3 mM. The fluorescence was read upon 570 nm of excitation and 585 nm of emission with a spectrofluorimeter (Varian Cary Eclipse, Agilent^®^). (ii) measurement of total H_2_O_2_: the same protocol was used except that root tips were first macerated before the reaction.

### 4.3. Transcriptomic Data Mining for CIII Prx Genes Involved in Low pH Response

We analyzed public transcriptomic data to search for CIII Prx encoding genes potentially involved in low pH response. The data set NASC 470 from Lager’s work [18] was downloaded at [55] using Expression Console™ 1.4.1.46 [56] to build an edited Microsoft Excel sheet [57]. The mean of the log2(value) for each condition (*n* = 3) was calculated as well as the ratio of absolute expression at pH 4.5 versus control at pH 6. The 73 *CIII Prx* genes [37] were searched for within the transcriptomic data using their probeset ID allowing the identification of ambiguous and non-ambiguous *CIII Prxs* [58]. Absolute heat map was drawn for the expression values (red to yellow to grey) with an arbitrary threshold value set as 5. Absolute heat map was drawn for ratio of absolute expression (blue to yellow). 

### 4.4. Evaluation of Cell Death

Cell death was evaluated by probing roots with Evans blue that can penetrate dead cells that lost membrane selectivity [59]. After pH treatments the seedlings were stained with Evans blue aqueous solution (0.25% w/v) for 15 min. Then, they were washed three times for 5 min each with distilled water and bright field images were taken using a Zeiss Axio Zoom V16 stereomicroscope. All procedures were performed in glass Petri dishes taking care of avoiding damages or root dehydration.

### 4.5. Image Analysis

To obtain semi-quantitative data, the images of Evans blue staining or of CIII Prx activity in roots were analyzed using the ImageJ software [60]. These images were used to draw the contour of each root tip, reaching 500 µm and 350 µm from the root tip for Evans blue staining and CIII Prx activity, respectively, constituting the regions of interest (ROIs). In both cases, the mean gray values of these ROIs were obtained. From each of these values, the mean gray value of the background of the corresponding bright-field image was subtracted to compensate variations in the light intensity between each image. The results were expressed as pixel intensity of the mean gray value. Thus, the increase of pixels intensity was straightforward interpreted as an increase in cell death or increase in total CIII Prx activity.

### 4.6. Whole Mount In Situ mRNA Hybridization

The protocol described in Hejatko’s work [61] was followed using the solutions described in detail in Francoz et al. [58] using 5 day old seedlings. The samples (10–20 seedlings) were processed in 0.95–1 mL solution/condition in 24 well sterile plate or in 1.5 mL RNase free microtubes for the hybridization step.The following minor modifications were introduced: use of Roti^®^ Histol (Carl Roth, Karlsruhe, Germany) for sample permeabilization, replacement of heparin with dextran sulfate in the hybridization solution. The digoxigenin-labelled riboprobes for detection of *AtPrx62* or *AtPrx42* were previously described [52]. The key parameters were as follows: (i) 125 µg mL^−1^ proteinase K for prehybridization; (ii) hybridization with a digoxigenin-labelled riboprobe at a final concentration of 50 ng/kb/mL for 16 h at 55 °C; (iii) immunodetection of hybridized probes with 1:2000 diluted anti-digoxigenin-alkaline phosphatase (AP) Fab-fragments (Roche, Basel, Switzerland) [52]; (iv) BCIP-NBT reaction for 48 min in the dark; (v) final mounting of samples in 50% (w/v) glycerol; (vi) and microscope analysis using a slide Nanozoomer slide scanner (Hamamatsu, Shizuoka, Japan) to produce whole slide scan at 20× resolution with five 10 µm-z scans to ensure finding the correct focus for all samples. The scans were analyzed using NDP view (Hamamatsu) and the images were directly extracted from the viewer to assemble the Figure. 

### 4.7. Statistical Analysis

We conducted randomized experiments. For each parameter analyzed at least three independent experiments were performed. Each biological replicate was composed of at least 10 seedlings. Means were compared by analysis of variance (ANOVA), followed by Duncan’s test. Only two means were compared by Student’s *t*-test at the 5% significance level.

## Supplementary Materials

Supplementary materials can be found at https://www.mdpi.com/1422-0067/21/19/7191/s1. Supplementary Table S1: Transcriptomics data mining for CIII Prx multigenic family expression following low pH treatment of A. thaliana roots reveals AtPrx62 as the most promising candidate gene for involvement in low pH-induced cell death in roots. Supplementary Figure S1: Identification of root zones in 5 day old seedlings of A. thaliana (Col-0) based on activity of CIII Prx and O2^•−^ distribution. Supplementary Figure S2. Measurement of H2O2 in root tips of A. thaliana (Col-0) after treatment at pH 5.8 or 4.6. Supplementary Figure S3. CIII Prx activity in A. thaliana Col-0 and atprx62-1 (KO mutant) treated at pH 5.8 or 4.6 for 2 h. Supplementary Figure S4. NBT reaction showing O2^•−^ distribution in roots of A. thaliana Col-0 and atprx62-1 (KO mutant) treated at pH 5.8 or pH 4.6 for 2 h. Supplementary Figure S5. Biological replicates for whole-mount in situ hybridization.

## Abbreviations

CIII Prx	Class III Peroxidase
CW	Cell Wall
CWD	Cell Wall Disturbance
CWI	Cell Wall Integrity
EZ	Elongation zone
MZ	Meristematic zone
PCD	Programmed Cell Death
ROS	Reactive Oxygen Species
TZ	Transition zone
