# Supplementary material for: The Class III Peroxidase Encoding Gene AtPrx62 Positively and Spatiotemporally Regulates the Low pH-Induced Cell Death in Arabidopsis thaliana Roots
Source: Int J Mol Sci. 2020 Sep 29;21(19):7191. doi: 10.3390/ijms21197191 (PMC7582640; doi:10.3390/ijms21197191)
Supplement: Supplementary file 1 [file ijms-21-07191-s001.zip › Graças et al_Sup data_R1.doc]

**The Class III peroxidase encoding gene *AtPrx62* positively and spatiotemporally regulates the low pH-induced cell death in *Arabidopsis* *thaliana* roots**

**Supplemental Figures**

**Supplemental Figure S1.** **Identification of root zones in 5 day-old seedlings of *A. thaliana* (Col-0) based on CIII Prx activity and superoxide (O_2_^•−^) distribution.**

Roots were treated at pH 5.8 or 4.6 for 3.5 h. **(A)** After treatment at pH 5.8, reaction with Nitroblue tetrazolium shows that the highest O_2_^•−^ concentration (strong purple) is located in MZ in agreement with Dunand et al (2007). **(B)** After treatment at pH 5.8, reaction with guaiacol and hydrogen peroxide (H_2_O_2_) shows that CIII Prx activity (brown color) is localized in EZ and TZ, but not in MZ. The size of elongating cells can be observed. **(C)** After treatment at pH 4.6, CIII Prx activity is localized in EZ and TZ and spreads to MZ stele. **(D)** After treatment at pH 4.6, Evans blue staining shows that cell death occurs in MZ, TZ and early EZ, below the zone of root hair differentiation. The tip-growing root hairs died upon low pH (not shown). MZ: Meristematic zone, TZ: Transition zone, EZ: Elongation zone. Scale bar: 200 µm.

**Supplemental Figure S2.** **Measurement of H_2_O_2_ in root tips of *A. thaliana* (Col-0) after treatment at pH 5.8 or 4.6.**

Bars are standard error of 3 independent experiments. Each treatment was composed of 10 seedlings. Note that no significant differences were observed between H_2_O_2_ levels among the treatments with this method measuring the H_2_O_2_ that diffused freely through the apoplast. No significant differences between H_2_O_2_ levels were neither observed when root tips were first macerated before the reaction to measure total H_2_O_2_ (data not shown). See methods for details.

**Supplemental Figure S3. CIII Prx activity in *A. thaliana* Col-0 and *atprx62-1* (KO mutant) roots treated at pH 5.8 or 4.6 for 2 h.** CIII Prx activity (brown color) was visualized by reaction with exogenously applied guaiacol and H_2_O_2_. In Col-0 roots treated at pH 4.6, CIII Prx activity showed a strong signal from inner cells in the tip, most likely in stele cells. This staining appeared less intense in the *atprx62-1*. Scale bar: 200 µm.

**Supplemental Figure S4.** **NBT reaction showing O2^•−^ (purple) distribution in roots of *A. thaliana* Col-0 and *atprx62-1* (KO mutant) treated at pH 5.8 or pH 4.6 for 2 h**. Roots were treated at pH 5.8 or 4.6 for 2 h. The images are representative of at least 15 roots of each treatment. Scale bar: 200 µm.

**Supplemental Figure S5.** **Biological replicates for whole-mount *in situ* hybridization.**

Root tip images from 4 individual seedlings per hybridization condition were directly extracted from the Nanozoomer scans to illustrate the reproducibility of the hybridization patterns observed. Details for the samples (Col-0 or *atprx62-1* KO mutant 5 day-old seedlings), the pH treatment (2 h control pH 5.8 or 2 h stress pH 4.6), the antisense (AS) riboprobe and corresponding sense (S) negative control probes used to detect *AtPrx62* or *AtPrx42* transcripts used as a constitutively expressed control gene are directly labeled on the figure. The two images on the bottom right are examples of wide views of two Nanozoomer scans from which the root tip images were extracted. Bars: 100 µm except for two wide views on the bottom right: 1 mm.

Fixative: 4 % (w/v) paraformaldehyde, 15 % (v/v) DMSO () and 0.1 %(v/v) Tween-20 in 1X PBS.

) DRY BATH
